# Extended Duration Vascular Endothelial Growth Factor Inhibition in the Eye: Failures, Successes, and Future Possibilities

**DOI:** 10.3390/pharmaceutics10010021

**Published:** 2018-01-27

**Authors:** Michael W. Stewart

**Affiliations:** Department of Ophthalmology, Mayo Clinic School of Medicine, 4500 San Pablo Rd., Jacksonville, FL 32224, USA; stewart.michael@mayo.edu; Tel.: +904-953-2232; Fax: +904-953-7040

**Keywords:** age-related macular degeneration, diabetic macular edema, extended duration therapy, intravitreal injections, vascular endothelial growth factor

## Abstract

Vascular endothelial growth factor (VEGF) plays a pivotal role in the development of neovascularization and edema from several common chorioretinal vascular conditions. The intravitreally injected drugs (aflibercept, bevacizumab, conbercept, pegaptanib, and ranibizumab) used to treat these conditions improve the visual acuity and macular morphology in most patients. Monthly or bimonthly injections were administered in the phase III pivotal trials but physicians usually individualize therapy with *pro re nata* (PRN) or treat and extend regimens. Despite these lower frequency treatment regimens, frequent injections and clinic visits are still needed to produce satisfactory outcomes. Newly developed drugs and refillable reservoirs with favorable pharmacokinetic profiles may extend durations of action and require fewer office visits. However, we have learned from previous experiences that the longer durations of action seen in strategically designed phase III trials often do not translate to less frequent injections in real-life clinical practice. Unfortunately, long-acting therapies that produce soluble VEGF receptors (encapsulated cell technology and adenovirus injected DNA) have failed in phase II trials. The development of longer duration therapies remains a difficult and frustrating process, and frequent drug injections are likely to remain the standard-of-care for years to come.

## 1. Introduction

The discovery of vascular endothelial growth factor (VEGF) [1,2] and the subsequent recognition of its critical role in the pathogenesis of several chorioretinal vascular conditions constitute the most important advances in ophthalmology over the past 30 years. Strong evidence correlates the development of both neovascularization and macular edema in the two most common causes of blindness in industrialized nations—neovascular age-related macular degeneration (nAMD) and diabetic retinopathy (DR)—with the upregulation of VEGF [3]. Furthermore, disease severity frequently correlates with intraocular VEGF concentrations, thereby making VEGF a logical target for therapeutic intervention. 

Soon after VEGF was discovered and sequenced, the production of inhibitory molecules began [4]. Thus far, five VEGF-neutralizing molecules (pegaptanib, Macugen^®^, Bausch & Lomb, Bridgewater, NJ, USA; ranibizumab, Lucentis^®^, Genentech, S. San Francisco, CA, USA/Roche, Basel, Switzerland; aflibercept, Eylea^®^, Regeneron, Tarrytown, NY, USA; conbercept, Chengdu Kanghong Pharmaceutical Group, Chengdu, China; and bevacizumab, Avastin^®^, Genentech, S. San Francisco, CA, USA/Roche, Basel, Switzerland) have been used to treat ophthalmologic conditions, though only the first three have received United States Food and Drug Administration (US FDA) approval for intraocular use. Intravitreal therapy usually begins with monthly injections (in accordance with package labeling) but most physicians will attempt to extend the time between injections as much as possible with either monthly *pro re nata* (PRN) or treat and extend strategies [5]. Treatment intervals for many patients cannot be extended beyond eight weeks [6], resulting in a large group of patients who require frequent injections for long periods of time. This large number of intravitreal injections burdens physicians and their staffs, and challenges patients’ compliance. Therefore, new, longer acting anti-VEGF medications and drug delivery systems are needed to improve outcomes, optimize compliance, and reduce the total cost of care.

This manuscript discusses extended duration anti-VEGF therapies that have been recently introduced, as well as those that are in various stages of development. 

## 2. Vascular Endothelial Growth Factor (VEGF) Physiology and Pharmacokinetics

VEGF was discovered independently by two research groups in 1989 [1,2] and its important role in both physiologic angiogenesis and pathological neovascularization was realized almost immediately. VEGF is actually a group of molecules that segregate into seven closely related families: VEGF-A, VEGF-B, VEGF-C, VEGF-D, VEGF-E, VEGF-F, and placental growth factor (PlGF) [7]. Each of the families is characterized by common, critical binding sequences, and most families contain multiple isoforms that share similar binding properties and biological actions. 

VEGF-A synthesis is upregulated in eyes with chorioretinal vascular conditions, including nAMD, diabetic macular edema (DME), and retinal vein occlusion (RVO) [3], and is believed to play a central role in the development of these conditions. Several in vivo models show that VEGF-A promotes the growth of choroidal neovascular membranes [8] and produces retinal vascular lesions that resemble DR [9]. Evidence suggests that VEGF_165_ may be the most biologically active isoform because of its high tissue concentrations and 10-fold potentiation of activity through its interaction with the transmembrane co-receptor neuropilin-1 [10]. Most VEGF inhibitory molecules block the receptor binding region (amino acids 81–92) of VEGF-A isoforms, whereas pegaptanib interacts with the heparin binding region (amino acids 110–165) of VEGF_165_. Research suggests that VEGF-B, VEGF-C, VEGF-D, and PlGF may also contribute to pathologic ocular angiogenesis in humans but their relative contribution is not known [11,12].

Increased VEGF synthesis by vascular endothelial cells, glia, pericytes, Müller cells, retinal pigment epithelium (RPE) cells, and invading leukocytes [13,14] results from tissue ischemia and inflammation [15,16]. Cells throughout the retina and choroid respond to increased VEGF concentrations but the primary targets are retinal and choroidal vascular endothelial cells [17].

VEGF-A has a short half-life of 30 min in the eye and serum, and homeostatic concentrations are generally low (approximately 9 ng/mL) [18]. Some systemic conditions increase serum VEGF concentrations but chorioretinal vascular conditions produce insufficient VEGF to meaningfully change serum levels.

## 3. Currently Available Therapies

Several anti-VEGF drugs have been developed exclusively for ocular use or, in the case of bevacizumab, are used off-label for chorioretinal vascular conditions. Peak clinical efficacies of these drugs (except for pegaptanib) are similar and though product labels describe different injection intervals (monthly or every two months) the differences in their duration of action are on the order of only days. Currently available drugs, recently failed therapies, and drugs and systems under development are listed in Table 1.

### 3.1. Pegaptanib

Pegaptanib (molecular weight (MW) of 50 kDa), an aptamer to VEGF, was the first ocular drug approved for the intravitreal treatment of neovascular age-related macular degeneration (nAMD). Clinicians hoped that q6week treatment with pegaptanib would improve best corrected visual acuity (BCVA) but in most eyes it only decreased the rate of vision loss by approximately one half [19]. Its use dropped significantly when more potent anti-VEGF drugs were introduced and pegaptanib is rarely used today.

### 3.2. Bevacizumab

Bevacizumab is a full-length, recombinant, humanized, monoclonal antibody (MW of 149 kDa) that binds all isoforms of VEGF-A. It was developed and approved for the intravenous treatment of several advanced solid tumors (colorectal carcinoma, non-small cell lung carcinoma, renal cell carcinoma, glioblastoma, and breast cancer, though this approval was rescinded in 2011) [20].

Single injections of bevacizumab were first given to patients with nAMD and macular edema due to a central retinal vein occlusion (CRVO) in 2005 [39,40], and within six months off-label use of bevacizumab had become the accepted standard-of-care treatment of chorioretinal vascular conditions. Hundreds of ocular disease studies have established bevacizumab’s efficacy and safety, though the best evidence comes from the Comparison of Age-related Macular Degeneration Treatment Trials (CATT) for nAMD and the Diabetic Retinopathy Clinical Research Network Protocol T trial for DME [6,21].

The use of bevacizumab varies among countries due to regulatory restrictions, reimbursement policies, and availability of safely compounded drug. Because physicians have accumulated extensive clinical experience with bevacizumab and are able to acquire it inexpensively, bevacizumab remains the most commonly used anti-VEGF drug in the United States.

### 3.3. Ranibizumab

Ranibizumab is a recombinant, humanized, monoclonal antibody fragment (Fab with MW of 48 kDa) that binds all isoforms of VEGF-A [4]. It has been approved by the United States Food and Drug Administration (USFDA) for the treatment of nAMD (2006), DME, DR, macular edema due to vein occlusions, and choroidal neovascular membranes (CNVM) associated with high myopia [22]. 

Following completion of the phase III MARINA and ANCHOR trials, ranibizumab was approved for the monthly treatment of nAMD and subsequently for PRN treatment. The CATT trial reported that PRN treatment is non-inferior to monthly treatment for nAMD [6] though pooled data from CATT and IVAN suggest that PRN is inferior to monthly injections. Ranibizumab is approved for the monthly treatment of DME, but after one year of intensive treatment in the Diabetic Retinopathy Clinical Research (DRCR).net Protocol I trial, less frequent injections are needed during subsequent years [41]. 

Because ranibizumab was the first approved intravitreal anti-VEGF drug (after pegaptanib), it became the standard against which other drugs have been compared in most randomized, controlled trials. These trials have included CATT [6], IVAN [42], and the other national AMD trials; the VIEW 1 and 2 trials (nAMD) [43]; CEDAR and SEQUOIA (nAMD); and DRCR.net Protocol T (DME) [21].

### 3.4. Aflibercept

Aflibercept is a recombinant fusion protein (MW of 115 kDa) consisting of the natural (all human) extracellular ligand binding sequences of VEGFR1 (domain 2) and VEGFR2 (domain 3) attached to the Fc portion of an IgG molecule [24]. Aflibercept is approved for the treatment of nAMD, DME, DR, and macular edema due to RVO [23].

The three-dimensional configuration of aflibercept enables it to simultaneously bind both sides of the VEGF dimer in a “two-fisted grasp”. This results in a higher binding affinity for VEGF_165_ (*k_D_* = 0.45 pM) compared to ranibizumab (*k_D_* = 46–172 pM) and bevacizumab (*k_D_* = 58–1100 pM) [44]. Rabbit studies suggest that aflibercept has a slightly longer intravitreal half-life that either bevacizumab or ranibizumab but head-to-head human studies have not been performed [45].

Peak efficacy of aflibercept in patients with nAMD is similar to that of ranibizumab but the duration of action is slightly longer [46]. Though aflibercept is approved for q8week dosing (compared to monthly for ranibizumab), its duration of action exceeds that of ranibizumab by only five to seven days. So despite the fact that the phase III trials suggested that aflibercept could be equally effective with only half the dosing frequency of ranibizumab, clinical use suggests that the difference is considerably shorter.

Ziv-aflibercept (Zaltrap^®^, Regeneron, Tarrytown, NY, USA) is the intravenous formulation of aflibercept that is used to treat advanced colorectal carcinoma. Small series of patients with nAMD, DME, and RVOs have responded well to intravitreal ziv-aflibercept with excellent improvements in macular morphology and visual acuity [47]. Head-to-head studies with aflibercept have not been performed, but the two molecules will likely perform comparably, though the lower dose of ziv-aflibercept (1.25 mg vs. 2 mg) may provide a slightly shorter duration of action.

### 3.5. Conbercept

Conbercept (KH902, Chengdu Kanghong Biotech Co., Sichuan, China) is a recombinant, fusion protein (MW of 143 kDa) that contains the second immunoglobulin (Ig) binding domain from VEGFR1, the third and the fourth binding domains from VEGFR2, and the Fc region of human IgG. Like aflibercept, conbercept acts as a soluble, decoy receptor [24,25] that binds all isoforms of VEGF-A, VEGF-B, and placental growth factor. Conbercept has a high affinity for VEGF_165_ (*k_D_* = 0.77 pM) because the fourth Ig domain of VEGFR2 enhances the association rate of VEGF to the receptor [25]. 

At concentrations between 100 ng/mL and 100 µg/mL, conbercept is not cytotoxic to cultured human retinal vascular endothelial cells (hRVACs). Conbercept significantly suppresses glucose-induced migration and sprouting of hRVACs by downregulating the expression of phosphoinositide 3-kinase and inhibiting the activation of Src, Akt1, and Erk1/2 [48]. Four weeks after intravitreal injection, conbercept-treated diabetic rats had better retinal electrophysiological function, less retinal vessel leakage, and lower levels of PlGF, VEGFR2, PI3K, Akt, p-Akt, p-ERK and p-SRC than did Pbs or bevacizumab-treated rats [49]. The distribution of claudin-5 and occludin in the retinal vessels of diabetic rats treated with conbercept was smoother and more uniform than those of diabetic rats treated by Pbs or bevacizumab. 

Conbercept is approved in China for the treatment of nAMD and a phase III trial evaluating the efficacy of conbercept for the treatment of DME is currently enrolling patients. Conbercept trials within the United States are now being planned.

## 4. Therapies under Development

The currently available anti-VEGF drugs have significantly advanced our treatment of chorioretinal vascular conditions and have benefitted hundreds of thousands of patients, but injections must usually be administered every four to eight weeks and treatment often continues for years. The extended durations of action that were promised by the newer drugs have not concretized, since a wealth of clinical experience shows us that the differences among the drugs are far shorter than are suggested by the packaging labels.

Nevertheless, research continues with new drugs and delivery methods that developers hope will extend the clinical duration of action. Several of the most promising drugs and some of the recent failures are discussed below.

### 4.1. Abicipar Pegol

Abicipar pegol is a designed ankyrin repeat protein (DARPin) that binds all isoforms of VEGF-A. Its small size (MW = 34 kDa) would suggest a brief intraocular half-life, but pegylation (binding to a poly(ethylene) glycol moiety) may give it the pharmacokinetic characteristics of a much larger molecule (approximately 250–350 kDa) [50]. Abicipar has an intravitreal half-life of six days in rabbits and, in a small DME study of four eyes, of 13.4 days in humans [26]. Its strong binding affinity to VEGF_165_ (*k_D_* = 2 pM) also favors a long duration of action.

In dose escalation trials, a maximum tolerated dose of 4.2 mg was found, so investigators elected to develop the two-milligram dose. In the phase II PALM DME trial, abicipar injections every 8 or 12 weeks were non-inferior to monthly ranibizumab [51]. In the ongoing phase III nAMD CEDAR (NCT02462928) and SEQUOIA (NCT02462486) trials, q8week and q12week abicipar is being compared to monthly ranibizumab.

### 4.2. Brolucizumab

Brolucizumab is a single-chain, high binding affinity (*k_D_* for VEGF_165_ = 1.6 pM), antibody fragment currently being developed by Alcon/Novartis (Ft. Worth, TX; Basel, Switzerland) for the treatment of nAMD [52]. Its small size (MW = 26 kDa) allows for the injection (six milligrams) of 12–24 times as many molecules as with the other anti-VEGF drugs [27].

A phase II clinical trial compared brolucizumab to aflibercept in patients with nAMD. The trial’s primary objective was to compare the efficacy of six-milligram brolucizumab against two-milligram aflibercept with the primary endpoint being the mean change in BCVA from baseline to 12 weeks. Patients continued receiving q8week treatment until week 40, though brolucizumab patients were eligible for two q12week cycles. At week 12, BCVA gains with brolucizumab (+5.75 letters) were similar to those with aflibercept (+6.89 letters). Approximately 50% of brolucizumab patients were stable during the q12week cycles [53].

The phase III nAMD clinical trials, HAWK and HARRIER, were initiated in December 2014, with an enrollment goal of 1700 patients in more than 50 countries. These two-year, double-masked, multi-center trials randomize patients with untreated nAMD to one of two dosage intervals of brolucizumab, or aflibercept bimonthly. At the 48-week primary endpoint, mean BCVA gains in both brolucizumab arms were non-inferior to aflibercept. The majority of patients receiving six milligrams brolucizumab (57% and 52%) were maintained exclusively on q12week dosing [28].

### 4.3. Ranibizumab Port Delivery System

A refillable ranibizumab port delivery system is being co-developed by Genentech and ForSight Vision 4 to reduce the need for repeated intravitreal anti-VEGF injections. The preloaded implant is surgically implanted beneath the conjunctiva through a 3.2 mm scleral incision over the pars plana. The reservoir tip can be accessed easily in the office and refilled through the conjunctiva as needed. The device continuously releases ranibizumab into the vitreous between refills.

A phase I trial for patients with nAMD was performed in Latvia [29]. At baseline, the reservoir was implanted and eyes were given 500 µg of ranibizumab, 250 µg into the vitreous and 250 µg into the reservoir for sustained release. Additional refills were performed when indicated by optical coherence tomography (OCT) evaluation of disease activity. The primary endpoint was 12 months with an observation period that extended through 36 months. The primary objective of the study was safety assessment, with secondary objectives that included functional measurements.

Four of the patients suffered significant or serious adverse events (endophthalmitis, vitreous hemorrhage (2), and traumatic cataract) but three of these four had improved vision by the study’s endpoint. The average visual acuity gains for the cohort were +10 letters, 10 eyes (50%) gained at least three lines, and two (10%) lost at least three lines. The mean number of refills through 12 months was 4.8 per patient.

The multicenter, randomized, treatment-control, phase II LADDER trial will include 220 patients at 55 U.S. sites. Patients will be randomized 3:3:3:2 to receive one of three different ranibizumab implant doses or monthly 0.5 mg ranibizumab injections. Study enrollment was completed in October 2017 [30].

### 4.4. Gene Therapy

Avalanche Biotechnologies developed a viral delivery system (AVA-101) to induce long-term anti-VEGF receptor synthesis by the outer retina. An adenovirus vector inserts the DNA for a naturally occurring sFLT-1 (soluble VEGF receptor-1) into RPE cells. Infected cells synthesize and excrete the soluble VEGF inhibitory protein into the outer retina and choriocapillaris. 

In a phase IIa trial, 21 patients with nAMD received AVA-101, with 0.5 mg ranibizumab injected both at baseline and one month, and as rescue therapy when needed. Patients underwent core vitrectomy and subretinal injection of AVA-101 adjacent to the macula at day seven. Evaluations were performed monthly and patients were eligible for rescue ranibizumab therapy based on pre-specified criteria. Eleven control patients received only 0.5 mg ranibizumab monthly.

At the 52-week endpoint, mean improvement in BCVA was +2.2 letters in the AVA-101 group compared to −9.3 letters in the control group [31]. These differences were statistically significant. Mean center point thickness improved by −27 µm in the AVA-101 group and −85 µm in the control group. There were no serious ocular adverse events in the AVA-101 group and no systemic safety signals were noted. All patients in the AVA-101 group that were phakic at baseline developed cataracts and three (14%) developed moderate vitreous hemorrhages. Gene therapy was well tolerated by patients but the technology failed to provide a complete or durable anti-VEGF response.

Though AVA-101 produced superior BCVA changes compared to the control group, the overall performance of the AVA-101 group was disappointing. Soon after the phase IIa trial results were announced, Avalanche decided not to proceed with phase IIb trials [54].

### 4.5. Encapsulated Cell Technology

Encapsulated cell technology (ECT) uses immortalized RPE cells that have been programmed to over-synthesize a specified biochemical product, and packages them in a cylinder lined by semi-permeable membranes that allow ingress of nutrients and egress of the synthesized product. The membrane prevents outward migration of the modified cells while shielding them from the body’s immune system. The 10 mm long cylinder is surgically implanted through the pars plana and is sutured to the sclera.

Trials with ciliary neurotrophic factor (CNF) production have been completed in eyes with retinitis pigmentosa and atrophic AMD [32]. Pharmacokinetic analyses showed that the half-life of CNF production was 54 months and the ECT cylinder was well tolerated. Unfortunately, the trials failed to meet their primary therapeutic endpoints. 

Phase I trials with a cylinder that produces a high-affinity VEGF binding protein similar to aflibercept have been completed. A multi-center phase II trial compared a higher dose, anti-VEGF implant against ranibizumab therapy. The trial was discontinued early because a larger number of patients than expected required intravitreal rescue injections [33]. No further nAMD trials have been announced but Neurotech continues to develop the platform for other retinal vascular conditions such as macular telangiectasia.

### 4.6. Colloidal Carriers

Injections of some liposomal drug formulations have shown promise including early work with anti-VEGF agents. In experimental models, in vitro release of ranibizumab from negatively charged liposomes was exhausted at two days, whereas ex vivo transport across sclera (simulating a subconjunctival injection) occurred in a linear manner for seven days [34]. This suggests that sclera acts as a classic membrane that allows the diffusion of liposomal-formulated ranibizumab and raises the possibility that subconjunctival injections could serve as long-acting depots. These results differ from those reported by Kim et al. [55] in which poly lactic-co-glycolic acid nanoparticles and liposomes do not facilitate drug diffusion across sclera. A steep concentration gradient created by the thick sclera, Bruch’s membrane-choroid, and retinal pigment epithelium results in low drug concentrations within the retina. 

### 4.7. Pump Delivery

Microelectromechanical system (MEMS) technology is a miniaturized system that is currently used in insulin pumps to deliver drug to tissues. The Posterior MicroPump Drug Delivery System (PMP, Replenish Inc., Pasadena, CA, USA) using MEMS technology is implanted on the sclera, similar to placement of a glaucoma drainage device, to deliver drug into the eye. Long-term safety after implantation into animal eyes has been demonstrated [35,36] as the PMP reliably delivered 100 programmed doses of an anti-VEGF drug (equivalent to over eight years of therapy). The PMP was well tolerated by 11 patients with DME over three months, with no cases of endophthalmitis or strabismus [37].

### 4.8. Topical Therapy

PanOptica, Inc. is developing a topical anti-VEGF medication (PAN-90806) for the treatment of nAMD and proliferative diabetic retinopathy (PDR). In animal models, pharmacokinetic measurements show excellent drug concentrations in the central retina and choroid as long as 17 h after administration. Control of leakage and bleeding from choroidal neovascular membranes was comparable to that achievable with intravitreal anti-VEGF antibodies, but with minimal systemic exposure to the drug.

In a phase II trial with 50 treatment-naïve nAMD patients, an independent panel of experts judged that PAN-90806 showed promise as a therapeutic agent [38]. Approximately 45–50% of treated patients experienced improvements in vascular leakage, retinal morphology, and vision. No systemic adverse events were noted and ocular surface irritation due to the eye drops reversed when therapy was discontinued. PanOptica plans to investigate higher doses in a phase I/II nAMD trial, and a phase I trial for the treatment of PDR is underway. 

## 5. Discussion

The quest for longer duration anti-VEGF therapies continues along several fronts with the injectable drugs abicipar and brolucizumab most likely to achieve US FDA approval. Each drug may be shown to be effective as q12week therapy—roughly half of the brolucizumab patients were sustained on q12week injections—but the importance of such a finding is not clear. Control groups in the phase III trials were treated with q4week ranibizumab and q8week aflibercept but neither of these drugs was tested in a q12week arm. Therefore, true head-to-head comparisons of these control drugs to abicipar and brolucizumab have not been performed with similar injection frequencies. 

In the VIEW trials, q4week ranibizumab was compared to q8week aflibercept during the first year. Aflibercept-treated patients experienced comparable improvements in BCVA and edema at 52 weeks compared to ranibizumab and was approved for q8week therapy (compared to q4week for ranibizumab). However, when patients received PRN (with 12-week cap) injections in the second year of the trials, aflibercept-treated patients received a mean of 4.2 injections, compared to 4.7 for ranibizumab. This difference in durations of action has been estimated to be five to seven days and post-approval experience also suggests that the difference is small. It is reasonable to suspect that post-approval differences with abicipar and brolucizumab will also be disappointingly small.

The quest for a single application, long-term anti-VEGF therapy has been disappointing. Encapsulated cell technology and adenovirus-mediated gene therapy are exciting technologies, but both failed to perform adequately in phase II trials and neither developer will pursue phase III anti-VEGF trials.

The ranibizumab port delivery system allows for trans-conjunctival (as opposed to intravitreal) injections as needed. However, since the phase I trial required a mean of 4.8 refills over the course of 12 months, this does little to decrease the frequency of clinic visits or injections. Unless the new dosing arms in the phase II trials decrease the number of refills, many physicians will likely continue with PRN and treat and extend regimens since they have comparable treatment burdens.

The use of eye drops does not constitute long-duration therapy but some patients will prefer self-administering drops when coupled with infrequent visits to the clinic. Eye drops effectively treat many anterior segment conditions and experimental CNVM in rats, but drops do not effectively treat retinal disorders in humans. Because topically delivered medications must pass through cornea, conjunctiva, sclera, uvea, and vitreous to reach the retina, the molecule must be small. Therefore, antibody-related macromolecules would be ineffective in eye drop form.

We have been fortunate in identifying VEGF as a pivotal molecule in the pathogenesis of chorioretinal vascular conditions, but just as the search for additional molecular targets has been disappointing, our attempts to significantly extend the duration of action of anti-VEGF therapy has met with more failures than successes. Despite ongoing research, it remains likely that frequent injection of anti-VEGF drugs will remain the standard-of-care for several years to come.

## Figures and Tables

**Table 1:** This table lists the currently available anti-VEGF drugs, several that have failed clinical trials, and others that are in various stages of development. Additional information includes regulatory approvals and comments on drug characteristics, pharmacokinetics, preclinical studies, and clinical trials. AMD: age-related macular degeneration; DME: diabetic macular edema; DR: diabetic retinopathy; RVO: retinal vein occlusion; VEGF: vascular endothelial growth factor; PlGF: placental growth factor; CNVM: choroidal neovascular membrane; RPE: retinal pigment epithelium; BCVA: best corrected visual acuity.

**Currently Available Drugs**		
**Drug**	**Approvals**	**Comments**
Pegaptanib	Neovascular AMD	Binds to VEGF_165_ Poor efficacy [19], used rarely
Bevacizumab	Advanced carcinomas [20] Off-label for all ophthalmic use	Recombinant, humanized, murine antibody to VEGF-A National Eye Institute sponsored studies have established effectiveness for neovascular AMD [6], DME [21], and RVOs Inexpensive dose cost after compounding Most commonly used intraocular anti-VEGF drug in the United States
Ranibizumab	Neovascular AMD, DME, DR, Macular edema due to RVOs, Myopic CNVM [22]	Recombinant, humanized, murine antibody fragment (Fab) to VEGF-A [4] Most thoroughly studied anti-VEGF drug
Aflibercept	Neovascular AMD [23], DME, DR, Macular edema due to RVOs	Completely human, fusion protein, soluble receptor [24] High affinity for VEGF-A, VEGF-B, and PlGF
Conbercept	Neovascular AMD (China only)	Similar structure and binding affinity as aflibercept [24,25] In phase III DME trial United States trials being planned
**Therapies Under Development or Recently Failed**		
**Drug**	**Technology**	**Comments**
Abicipar	Designed Ankyrin Repeat Protein (DARPin)	Pegylation may extend intravitreal half-life (estimated as 13.4 days in humans) [26] Phase III CEDAR and SEQUOIA nAMD trials have completed enrollment q8week and q12week experimental arms; control is q4week ranibizumab
Brolucizumab	Single strand, antibody fragment	Small size (26 kDa) allows for injection of large quantity of drug [27] Phase III nAMD trials recently completed 57% and 52% of eyes sustained with q12week injection intervals [28]
Ranibizumab Port Delivery System	Trans-scleral refillable drug reservoir	Reservoir is refilled via trans-conjunctival injection Phase I study showed +10 letter improvement in BCVA with average of 4.8 refills [29] Phase II LADDER trial underway with three different dose treatment arms [30]
AVA-101	Adenovirus vector Insertion of soluble VEGF-receptor DNA	Injected subretinally after vitrectomy BCVA changes were better than ranibizumab in phase II trial but both arms performed poorly with minimal decrease in edema [31]
NT-503	Encapsulated Cell Technology using immortalized RPE cells	Ciliary neurotrophic eluting device failed in dry AMD and retinitis pigmentosa trials [32] High dose (NT-503) device failed in phase II neovascular AMD trial [33] Currently being tested in patients with macular telangiectasia
Colloidal Carriers	Liposomal formulated ranibizumab	Liposomal formulation delays drug release Ranibizumab can cross sclera after subconjunctival depot [34]
Posterior Micropump Delivery System	Microelectromechanical Systems (MEMS) Technology	Same technology as in insulin pumps Safely delivered 100 injections in animal models [35,36] Three-month DME trial in humans was well tolerated [37]
PAN-90806	Small molecular weight drug	Formulated for eye drop delivery In animal models, found to produce high retinal concentrations 17 h later Judged to show therapeutic promise in small human nAMD study [38]
